# Enhancement of Ni–Zn ferrite nanoparticles parameters via cerium element for optoelectronic and energy applications

**DOI:** 10.1186/s11671-023-03921-6

**Published:** 2023-11-08

**Authors:** R. M. Kershi, A. M. Alshehri, R. M. Attiyah

**Affiliations:** 1 Physics Department, Faculty of Science, King Khalid University, Abha, Saudi Arabia; 2 Physics Department, Faculty of Science, Ibb University, Ibb, Yemen

**Keywords:** Ferrite nanoparticles, Crystallite size, Dielectric constant, Spectroscopic properties, AC conductivity, Optical energy gap

## Abstract

This work is concerned with fabricating ferrite nanoparticles of nickel–zinc with the chemical formula: Ni_0.55_Zn_0.45_Fe_2−*x*_Ce_*x*_O_4_, 0 ≤ *x* ≤ 0.011 by co-deposition technique and modifying their electrical, microscopic, spectroscopic, optical, electrical and dielectric properties as advanced engineering materials through doping with the cerium (Ce) element. XRD patterns displayed that the samples have a monophasic Cerium–Nickel–zinc (CNZ) spinel structure without other impurities for cerium concentration (*x*) ≤ 0.066. Both values of crystallite size and lattice parameters decrease from 33.643 to 23.137 nm and from 8.385 to 8.353 nm, respectively, with the increasing Ce ions substitution content from 0 to 0.066. SEM images indicate that grains of the fabricated compounds are smaller, more perfect, more homogeneous, and less agglomeration than those of the un-doped Ni–Zn nano-ferrites. The maximum intensity of first-order Raman spectral peaks (*E*_g_, F2g(2), A1g(2), and A1g(1)) of CNZ ferrite nanoparticles are observed at about (330, 475, 650, 695) cm^−1^, respectively, that confirms the CNZ samples have the cubic spinel structure. The direct and indirect optical energy bandgaps of CNZ samples have a wide spectrum of values from semiconductors to insulators according to cerium concentration. The results showed that the values of dielectric constant, dielectric loss factor, and Ac conductivity and the conductivity transition temperature are sensitive to cerium ions content. AC conductivity exhibited by the CNZ samples has the semiconductor materials behavior, where the AC conductivity increases due to temperature or doping concentration. The results indicate that Ni_0.55_Zn_0.45_Fe_1.944_Ce_0.066_O_4_ ferrite nanoparticles may be selected for optoelectronic devices, high-frequency circuits, and energy storage applications.

## Introduction

Ferrite spinel compounds at the nanoscale are of deep interest in a wide range of scientific and technological applications. This attention comes due to features of physicochemical parameters such as high chemical stability, electrical resistance, Curie temperature, mechanical hardness, and surface area. These materials are used in microwaves, digital tape, and recording media. In addition, they are highly selected for bio-ferrofluids, magnetic refrigeration sensors, drug delivery, detoxification, magnetic anti-cancer drugs, magnetic resonance imaging, magnetic cell separation, and various other biological and medical applications [1–5]. There are many factors such as the structure, morphology, composition, defects, dopants, and method of synthesis influencing and controlling the properties and applications of spinel ferrite compounds. The rare earth element is distinguished by its triple valence and large radius generally and tries to enter the ferrite compound at the expense of the ferric ions. Spinel ferrite compounds become more promising compounds for various applications when they are doping with a small concentration of rare earth elements.

The ionic radius and concentration of substituted rare earth element (RE) play an outstanding role in altering the properties of the ferrite compounds due to the occurrence of 4f (RE^3+^)–3d(F^3+^) couplings. The difference in the ionic radii between guest and host atoms leads to lattice strain which may result in the deformation or defect of the microstructure of spinel ferrite or form a secondary phase [6]. Hence, the concentration of the substituted rare earth ions in the cell structure has limited solubility in the spinel lattice due to their large ionic radii. There are a number of reports written by researchers talking about the effect of adding rare earth elements on the properties of spinel ferrite nano-ferrite compounds [7–13]. Previous studies illustrated that it can be produced single-phase spinel nickel ferrite compounds doped with holmium and with yttrium rare earth ions with solubility limits equal to 0.15 and 0.07, respectively, and the lattice constant of mentioned nickel ferrite compounds samples increases with increasing both rare earth elements. The erbium ions reduce the lattice constant and increase the size of the crystals of Li–Ni spinel ferrite compounds [14]. In another study, an increase in the lattice constant and a decrease in the crystallite size were achieved, with an increase in the cerium ions content in cobalt spinel ferrite [15]. The lattice constant of nickel–zinc spinel ferrite compounds increases with increasing cerium ions [16].

Ni–Zn ferrite compounds are inverse spinel ferrite compounds with features for use in high-frequency devices higher than other soft magnetic materials as it has a high electrical resistivity associated with fantastic ferromagnetic properties [17]. In fact, there are a large number of studies related to the properties of Ni–Zn ferrite spinel [18–23] and many studies related to the effect of adding rare earth ions on the properties of Ni–Zn ferrite [24–29] but as far as the authors know that there is no comprehensive study concerned with studying the effect of cerium ions on the properties of Ni–Zn ferrite nanoparticles, especially the spectral properties. Based on that, in this manuscript, CNZ ferrite nanoparticles were fabricated via the chemical coprecipitation technique to optimize the structural, microscopic, and spectroscopic characteristics. The fabricated compounds were studied using different characterization techniques including XRD, SEM, UV–Vis, FTIR, RAMAN, and RLC to study in deep of the impact of the cerium ions doping on the structural, morphological, optical, spectroscopic, electrical, and dielectric properties of nickel–zinc spinel ferrite nanoparticles. In addition to determine the solubility limit of cerium ions in the stoichiometric ratio of nickel–zinc ferrite when it is synthesized under specific preparation conditions of pH and temperature.

## Experimental techniques

### Synthesis method

The coprecipitation technique was used to prepare Ni–Zn spinel ferrite compounds doped with Ce rare earth ions according to the chemical formula: Ni_0.55_Zn0_.45_Fe_2−*x*_Ce_*x*_, 0 ≤ *x* ≤ 0.11. The coprecipitation technique is widely employed in the synthesis of ferrite compounds due to its simplicity, low cost, and low sintering temperature [30, 31]. High-purity and highly homogeneous products in the nanoscale are produced via this technique [30].

### Raw materials

The raw materials used to prepare CNZ spinel ferrite nanoparticles are ferric chloride [FeCl_3_·9 H_2_O], cobalt chloride [CoCl_2_·6H_2_O], zinc chloride [ZnCl_2_·6 H_2_O], and Cerium nitrate [Ce (NO_3_)_3_·9H_2_O]. In addition to, sodium hydroxide pellets, ammonia solution, and distilled water. The precursor salts were produced by Sigma-Aldrich and were of high analytical purity and were therefore used without further purification.

### Synthesis of CNZ ferrite nanoparticles

Ni(II), Zn(II), and Fe(III) metals and Ce(III) rare earth salts were mixed in weight ratios to produce CNZ ferrite nanoparticles. Use distilled water as a solvent for the mixture of balanced proportions of salts and the solution was placed on the magnetic stirrer for 3 h. Then, the sodium hydroxide and ammonia solutions was added to the salt solution dropwise with continuous rotation by a magnetic stirrer until the pH value of the mixture became 11. Then, the mixture was left at room temperature on the magnetic stirrer for 24 h. The aqueous solution containing the precipitated nanoparticles was then filtered and washed several times with deionized water to get rid of sodium salts and unwanted ions. After that, the washed nanopowder was ground for half an hour and dried in an oven at 85 °C for 3 days. After that, the dry powder was finely ground and sintered in an electric oven at 1050 °C for 3 h, then finely ground again for half an hour to be ready at that time to study the properties of the fabricated compound.

### Synthesis of CNZ nano-ferrofluids

Samples of nano-ferrous fluids to study their optical and spectroscopic properties in the ultraviolet–visible range using a UV–Vis spectrometer were prepared using the ultrasonic device for 35 min and a power of 50 watts.

### Characterizations measurements

To study the crystalline structure and phase and calculate the structural parameters of the fabricated CNZ nano-ferrite samples based on the diffraction of the X-ray incident on them, the samples were subjected to rays with a wavelength of 1.54 Å from an instrument model a Shimadzu X-600 Japan. A Thermo Nicolet 6700 FTIR spectrometer was used to examine the spectral properties of CNZ nano-ferrites and vibrational bands of metallic oxygen at their both tetrahedral and octahedral *V*_A_ and *V*_B_ sites at wavenumbers from 400 to 4000 cm^−1^. Nano-ferrofluids ferrite compounds were exposed to the beams of a JASCO V-570 spectrophotometer over the wavelength range of 190–800 nm for the purpose of determining the optical parameters. Raman spectra of the fabricated samples were taken out using a Horiba Jobin Yvon HR800 UV: Raman spectrometer, with an excitation wavelength (*λ*) of 633 nm, in the range 200–800 cm^−1^ at 300 K. The CNZ ferrite nanoparticles were pressed uniaxially using a hydraulic press in the form of disks with a diameter of 14 mm and thickness about of 3 mm. The pressed disks were polished and coated with silver paste for measurements of electrical and dielectric properties using LCR bridge meter (Agilent 4284 A Precision LCR Meter) in the frequency region from 0 to 1000 kHz at room temperature and in the range of 300–700 K.

## Results and discussion

### The structural properties

**Fig. 1:**
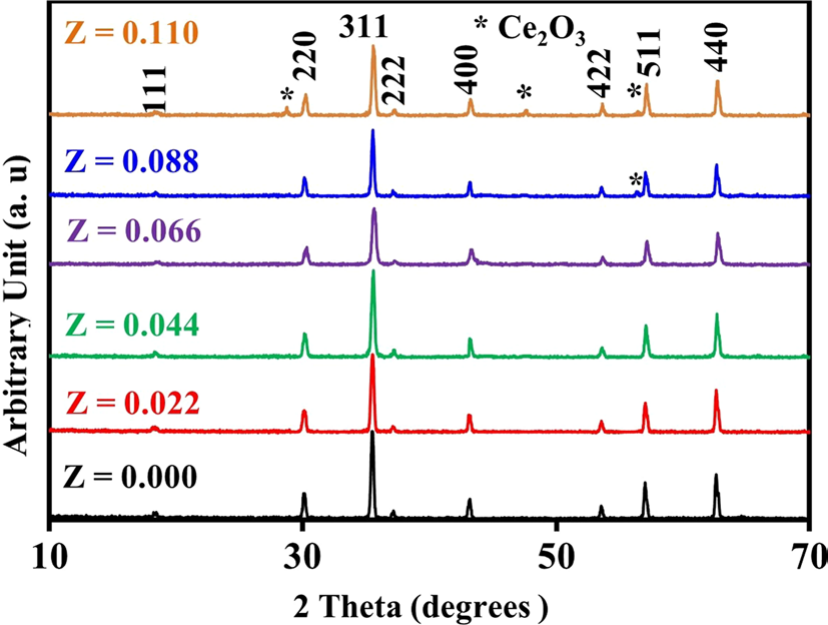
X-ray diffraction pattern of the CNZ ferrite nanoparticles as a function of cerium ions content

The X-ray diffraction patterns of the CNZ ferrite system prepared by the coprecipitation technique are depicted in Fig. 1. The diffraction peaks of the prepared samples showed that the samples with a doping concentration of cerium ions up to 0.066 have a pure cubic structure via the matching with JCPDS file No.: 08–0234. While the secondary phase of cerium oxide (Ce_2_O_3_) starts to arise for Ce ions content ≥ 0.066. Moreover, it is observed that the secondary phase increases with the cerium ion content (*x*) in Ni–Zn spinel ferrite compounds. The induced secondary phase with the rare earth substitution in Ni–Zn spinel ferrite samples agrees with several published research reports [26–42], and at the same time, this result contradicts many published research reports [43–50].

The solubility limit of the rare earth element in the structure of a ferrite compound depends on the type and amount of the rare earth element added and the preparation conditions as shown in Table 1. The rare earth element cerium has a large ionic radius (1.11 Å) [51] and is therefore difficult to insert into the lattice unit cell of Ni–Zn spinel ferrite. From Fig. 1, it can be concluded that the solubility limit of cerium ions in the Ni–Zn spinel ferrite unit cell is 0.066, after which the secondary phase begins to appear. There are several previous papers in Table 1 showing several values for the solubility limits of rare earth elements in Ni–Zn ferrite.

**Table 1:** The solubility limits of the rare earth elements in nickel–zinc ferrite compounds in many previous research papers in compared with this study

Ni–Zn–RE ferrites	Synthesis method	Sintering temperature (°C)	Solubility limit	References
Ni_0.5_Zn_0.5_Yb_*x*_Gd_*x*_Fe_2−*x*_O_4_	Sol–gel	700	0.04	[26]
Ni_0.5_Zn_0.5_Fe_2−*x*_Sm_*x*_O_4_	Combustion	480	0.1	[31]
Ni_0.65_Zn_0.35_Nd_*x*_Fe_2−*x*_O_4_	Coprecipitation	800	0.075	[28]
Ni_0.5_Zn_0.5_Fe_2−*x*_Nd_*x*_O_4_	Combustion	1100	0.04	[51]
Ni_0.5_Zn_0.5_Fe_2−*x*_Ce_*x*_O_4_	Combustion	800	0.02	[40]
Ni_0.55_Zn_0.45_Fe_2−*x*_Ce_*x*_O_4_	Coprecipitation	1200	0.066	This study

**Fig. 2:**
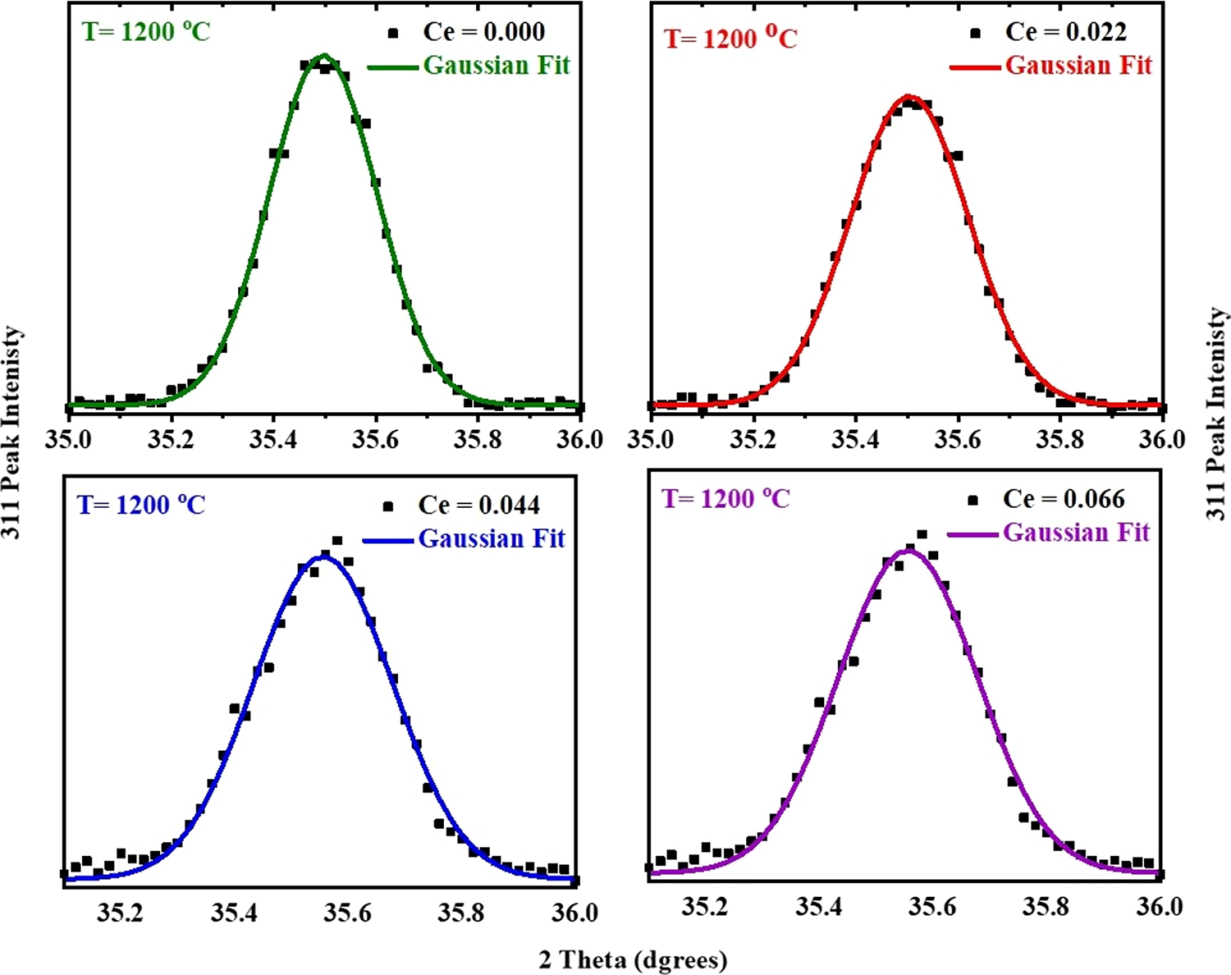
Gaussian fitting of 311 diffraction peak for the CNZ ferrite nanoparticles as a function of cerium ions content

**Fig. 3:**
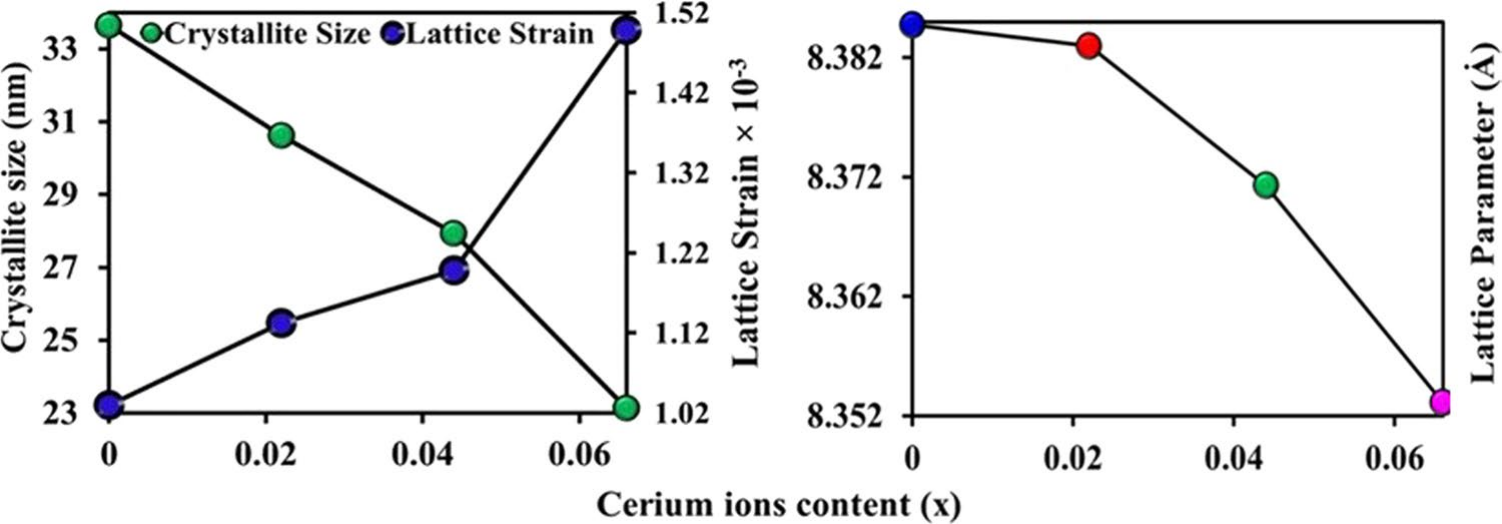
Lattice parameter, Crystallite size and lattice strain of the CNZ ferrite nanoparticles as a function of cerium ions content

Crystallite size values of CNZ ferrite nanoparticles as a function of cerium ion (*x*) content determined from the full width at half maximum (FWHM) of the main peak (311) using the Debye–Scherrer equation. The FWHM was calculated based on the Gaussian fitting of the 311 peaks of the different investigated CNZ nanoparticles as can be indicated in Fig. 2. The crystallite size of the prepared CNZ ferrite nanoparticles samples versus cerium ions concentration (*x*) is illustrated in Fig. 3. This figure indicated that the average crystallite size values of the samples decrease from 34 to 23 nm with increasing the concentration of cerium ions *x* from 0 to 0.066. Due to the process of forcing the large-radius cerium ion to shift the small-radius ferric ion, more activation energy to enter the octahedral sites (B) is required where the bond energy of Ce–O is higher in comparison to that of Fe–O [57] which means that the formation and growth of the Nickle-zinc ferrite doped with cerium ions (CNZ) need higher energy than that of pure Nickle-zinc ferrite (i.e., the CNZ ferrite compounds samples have higher thermal stability and can be used in high-thermal applications). Therefore, instead of occupying Fe^3+^ sites in the lattice, cerium ions Ce^3+^ enter into the interstitial sites of the lattice, which leads to inconsistency in the structure of the Ni–Zn samples and stimulates crystal anisotropy, which ultimately generates increased lattice strain with the increase of cerium ions as is evident from Fig. 3. In order for the crystalline anisotropy and lattice strain inside the crystal to remain in an equilibrium state, the crystal size decreases with the increase in the cerium ions content (*x*), and this is completely agreed with previous reports [7, 52].

The lattice parameter (*a*) was calculated using the relation $a = d_{hkl} \sqrt {h^{2} + k^{2} + l^{2} }$ and plotted in Fig. 3. In fact, it can be seen that the lattice parameter decreases from 8.385 to 8.353 nm with increasing Ce^3+^ ions content. This result is consistent with previous results [14, 50, 53]. The decrease in the lattice parameter with increasing cerium content (*x*) may be attributed to the deformation of the unit cell, thus a decrease in the alignment level of the lattice edges as a result of the addition of large cerium ions. In addition to a higher attraction force between O^−2^ and Ce^3+^ ions compared to that between O^−2^ and Fe^3+^ ions [44]. The variation of Volume of the unit cell (*V*), X-ray density (*ρ*_x_), bulk density (*ρ*_**B**_), porosity (*p*%), dislocation density (*ρ*_dis_) and specific surface area (*S*) of CNZ ferrite nanoparticles as a function of the cerium ions dopants concentration (*x*) is listed in Table 2. Table 2 illustrates that decreasing the volume of the unit cell (*V*) with increasing cerium ions content (*x*), this behavior is due to decreasing the lattice parameter (*a*) with *x* as can be observed from Fig. 3 where *V* = *a*^3^ for spinel ferrite with cubic structure. The unit cell of spinel ferrite has 8 tetrahedral sites (*A*) and each ion in these sites is surrounded by four oxygen ions and 16 octahedral sites (*B*), each ion in these sites is surrounded by six oxygen ions [54]. The jumping distance between the cations at the tetrahedral (*A*) and octahedral (*B*) interstitial sites in the CNZ structure was calculated based on the $L_{A} = \sqrt 3 a/4$ and $L_{A} = \sqrt 2 a/4$ relationships, respectively [54]. It can be seen that from the data of both the metal ions distances *L*_A_ and *L*_B_ in Table 2 decrease with the increase of Ce^3+^ cerium rare earth ions dopants in the investigated CNZ ferrite samples microstructure. The variation of metal ions distances follows up the values of lattice parameter (*a*).

**Table 2:** Volume of unit cell, X-ray density (*ρ*_*x*_), bulk density (*ρ*_B_), porosity (*p*%), dislocation density (*ρ*_dis_), specific surface area (*S*) and the hopping lengths (LA) and [LB] of CNZ ferrite nanoparticles as a function of cerium ions content

*L*_B_(Å)	*L*_A_(Å)	S (m^2^ g^−1^)	*ρ*_dis_ (10^10^ cm^−2^)	*p* %	*ρ*x (g cm^−3^)	*ρ*_B_ (g cm^−3^)	*V* (Å3)	Ce (*x*)
2.964	3.631	33.33	5.48	4.48	5.35	5.11	589.48	0.000
2.963	3.629	36.32	6.62	4.26	5.39	5.16	589.13	0.022
2.959	3.625	39.34	7.68	3.85	5.46	5.25	586.64	0.044
2.953	3.617	46.83	11.63	3.25	5.54	5.36	582.83	0.066

Theoretical density is calculated based on *ρ*_*XRD*_ = 8*MN*_*A*_*V* relation, where M is the molecular weight, and *N*_A_ is Avogadro’s number [54] while the bulk density was calculated using the relationship *m*/(*πr*^2^*t*) after pressing the samples in form disks with mass (*m*), radius ®, and thickness (*t*) [54]. The porosity (*p*%) was calculated using the following ratio *p*% = (1 − *ρ*_B_/*ρ*_XRD_)% [54] The relative increases in the bulk density may be attributed to the possibility of entry of rare earth cerium ions due to the widening of the lattice during the sintering process. During the cooling process, the lattice shrinks, leading to the removal of the pores and thus reducing the porosity of the prepared samples. The dislocation density (*ρ*_dis_) values are listed in Table 2. *ρ*_dis_ represents the number of defects in the crystals of the CNZ ferrite nanoparticles. The increase of *ρ*_dis_ with increasing concentration of the cerium ions in CNZ ferrite indicates that Ce^3+^ions increase the defects in Ni–Zn crystals which agree with the analysis of lattice strain and which in turn leads to an active role in reducing the crystallite size to achieve the equilibrium state for the fabricated compound. Moreover, Table 2 illustrates that the X-ray density (*ρ*_XRD_) increases with increasing Ce^3+^ ions concentration (*x*). This may be attributed to the decrease of unit cell volume and the increase of molecular weight of the samples with an increase of Ce^3+^ ions content (x), where the difference between the molecular weights of cerium ions (guest) and ferric ions (host) are 140.116 and 55.845 g. in mole-1, respectively. The specific surface area (S) of the prepared CNZ ferrite nanoparticles samples was determined with average crystallite size (D) and X-ray density using *S* = 6000/*D* *ρ*_XRD_ formula and found increases from 33.3 to 46.8 m^2^g^−1^ with increasing cerium rare earth ions as a response to the decrease of crystallite size.

### Morphological properties

**Fig. 4:**
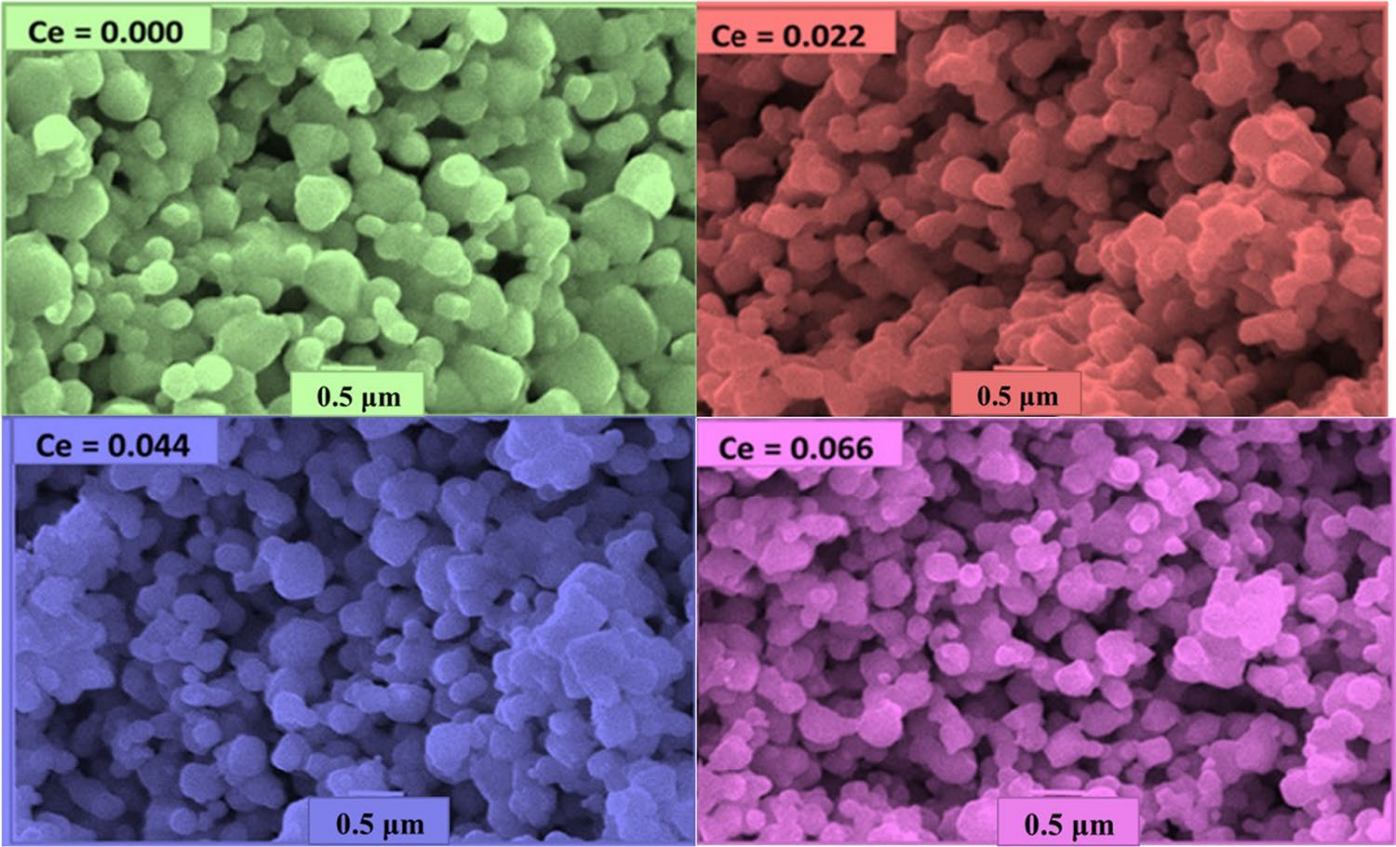
SEM image of the CNZ ferrite nanoparticles as a function of cerium ions content

**Fig. 5:**
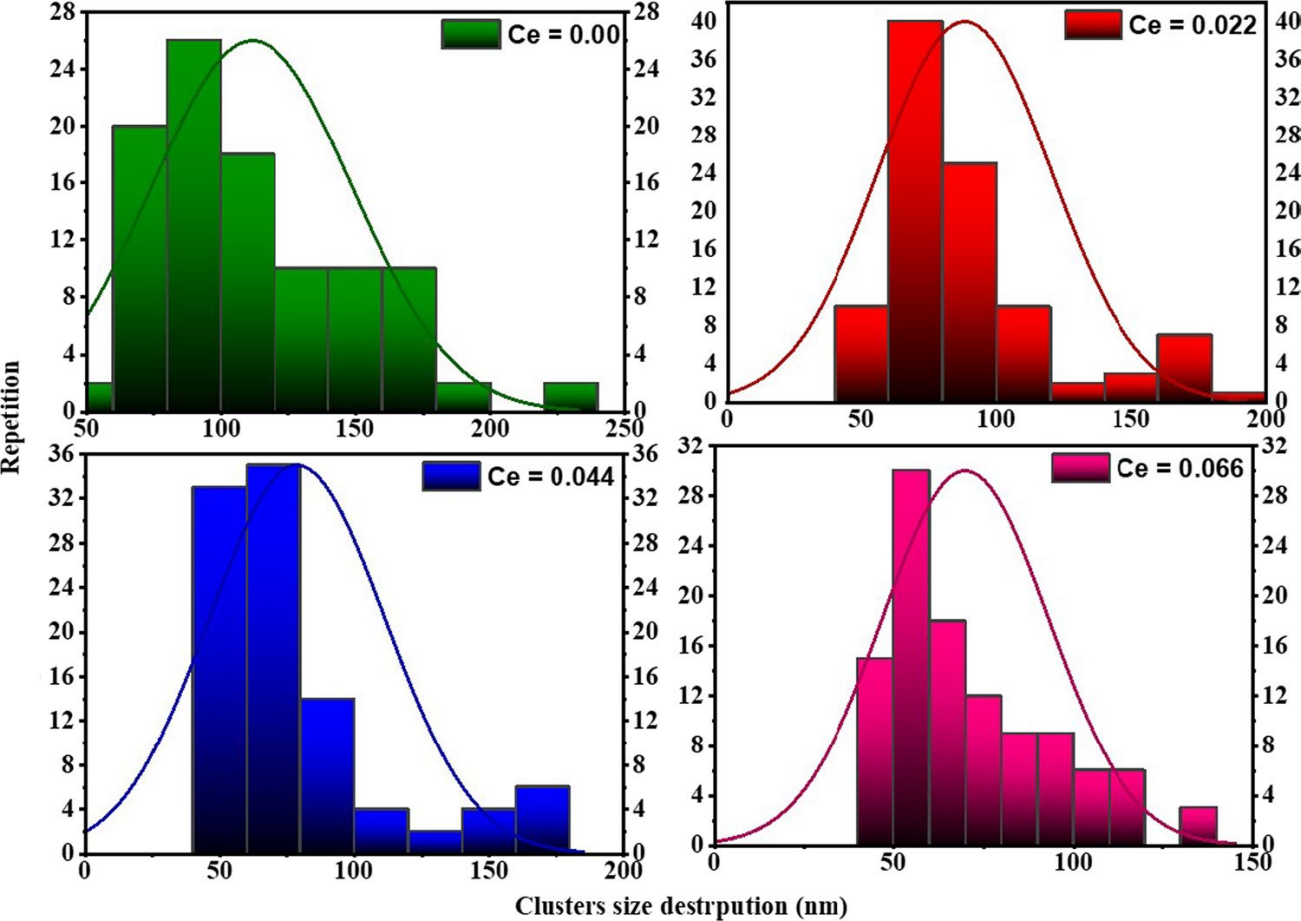
Particle size distribution of the CNZ ferrite nanoparticles as a function of cerium ions content

Figure 4 exhibits SEM images of the CNZ ferrite nanoparticles with different cerium ions content (*x*). It indicates that the particles of the fabricated CNZ ferrite compounds are fine, smooth, and semi-homogeneous spherical shapes. Moreover, the addition of cerium rare earth ions suppressed the growth of grains nuclei availably; the grain was smaller and, more perfect than that of the undoped Ni–Zn ferrite. The homogeneity of the particles of the prepared samples, as shown in the SEM images, confirms that the CNZ samples have the cubic spinel structure that was deduced from the results of the XRD data. There is no clear morphological variation in the prepared CNZ samples with different content of cerium ions. The particles of these samples are polydisperse and some of them agglomerated due to magneto-dipole interactions between their boundaries and the decrease of the agglomeration state of particles with increasing cerium rare earth ions can be notated also. The statistical analysis of particle size distribution (PSD) of the CNZ ferrite nanoparticles was analyzed using ImageJ software and plotted in Fig. 5. From the histograms can be glimpsed that the particle size decreases with increasing Ce^3+^ ions content. When ferric (Fe^3+^) ions in the octahedral sites of the spinel ferrite structure are replaced by cerium (Ce^3+^) ions, the lattice parameter (*a*) decreases and the distribution of cations at A and B sites rearranges which in turn lead to creating a lattice strain and internal stress. In other words, the internal lattice stress hinders the growth grains of CNZ ferrite nanoparticles and this produces particle size of CNZ samples smaller than that of pure Ni–Zn samples. This result indicates the effectiveness of Ni–Zn ferrite nanoparticles doped with a high concentration of Ce ions in water treatment applications due to their large specific surface area and the broken bonds at their surfaces. In addition to its semi-regular spherical shape, and the weakness of its agglomeration state.

### FTIR spectroscopic analysis

**Fig. 6:**
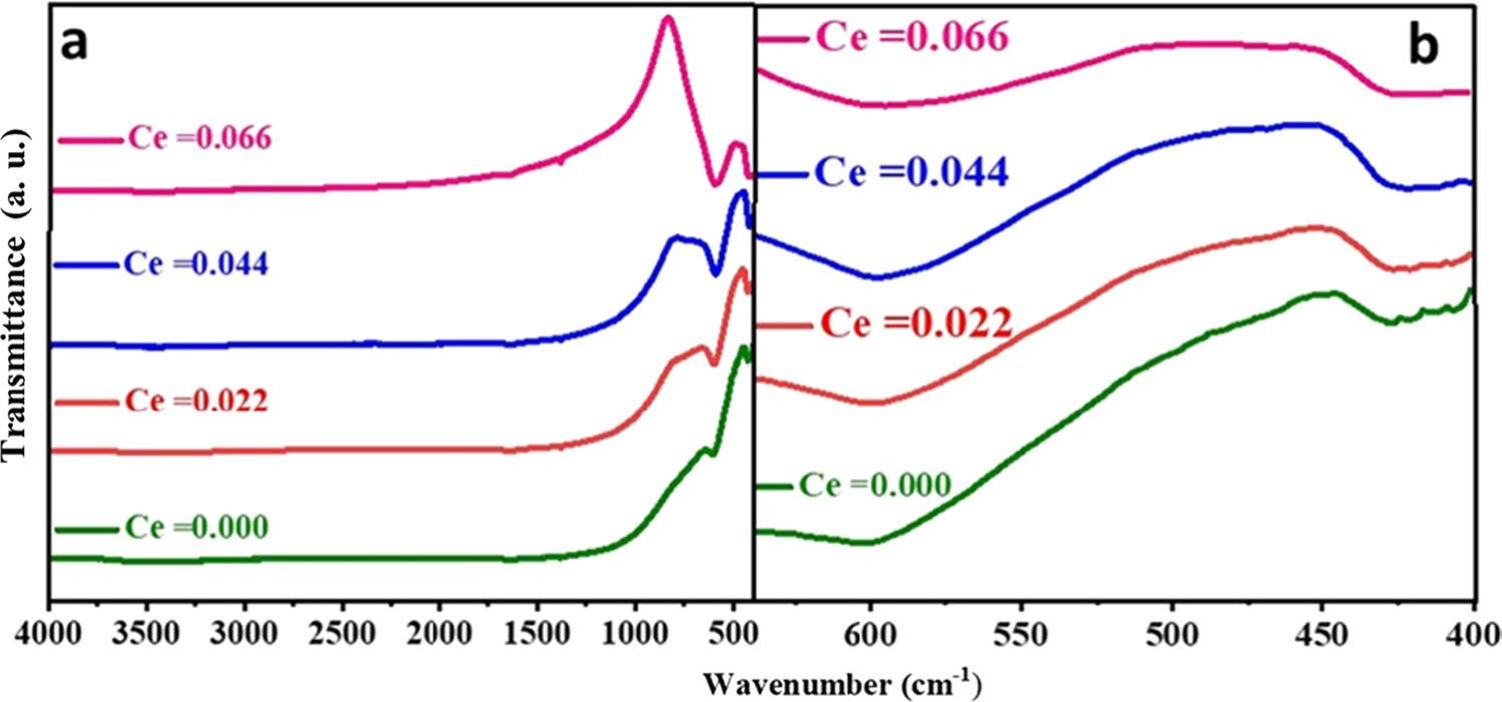
FTIR spectra of the CNZ ferrite nanoparticles as a function of cerium ions content in the range; **a** 400- 4000 cm^−1^ and **b** 400-650 cm^−1^

FTIR spectra of the fabricated CNZ spinel ferrite nanoparticles samples as a function of cerium ions content (*x*) are presented in Fig. 6 and the corresponding IR absorption bands of metal–oxygen in both tetrahedral and octahedral sites are listed in Table 3. The bands *V*_A_ and *V*_B_, which were considered as major bands are observed in the ranges 601.68–593.97 cm^−1^ and 428.11–418.28 cm^−1^, respectively. These vibrational bands *V*_A_ and *V*_B_ are represented as the stretches between oxygen anions and the metallic cations at the tetrahedral (*A*) and octahedral (*B*) sites, respectively. The variation in the FTIR spectra bands positions of the spinel ferrite depends on some factors such as the amount and type of dopants, the size and molecular weight of the compound, and the preparation method [53]. The shift to lower values of the vibrational bands *V*_A_ and *V*_B_ are noticed with Ce^3+^ ions content (*x*) in the Ni–Zn spinel lattice. The decrease of the vibrational wavenumbers *V*_A_ and *V*_B_ may be attributed to the larger value of the molecular weight of cerium ions than that for ferric ions in the octahedral sites and to the short value of *L*_A_ bonds in tetrahedral sites.

**Table 3:** The absorption bands (*ν*_A_ and *ν*_B_), and the optical band gaps (*E*_g_ (dir.) and *E*_g_ (indir.)) of CNZ ferrite nanoparticles as a function of ions content

Ce (*x*)	*E*_*g*(dir.)_ eV	υ_B_ (cm^−1^)	υ_A_ (cm^−1^)	*E*_*g*(indir.)_ eV
0.000	3.75	428.11	601.68	2.20
0.022	3.8	426.15	599.75	2.30
0.044	3.84	422.33	595.62	2.40
0.066	3.88	418.28	593.97	2.50

### Uv–Vis spectroscopic analysis

**Fig. 7:**
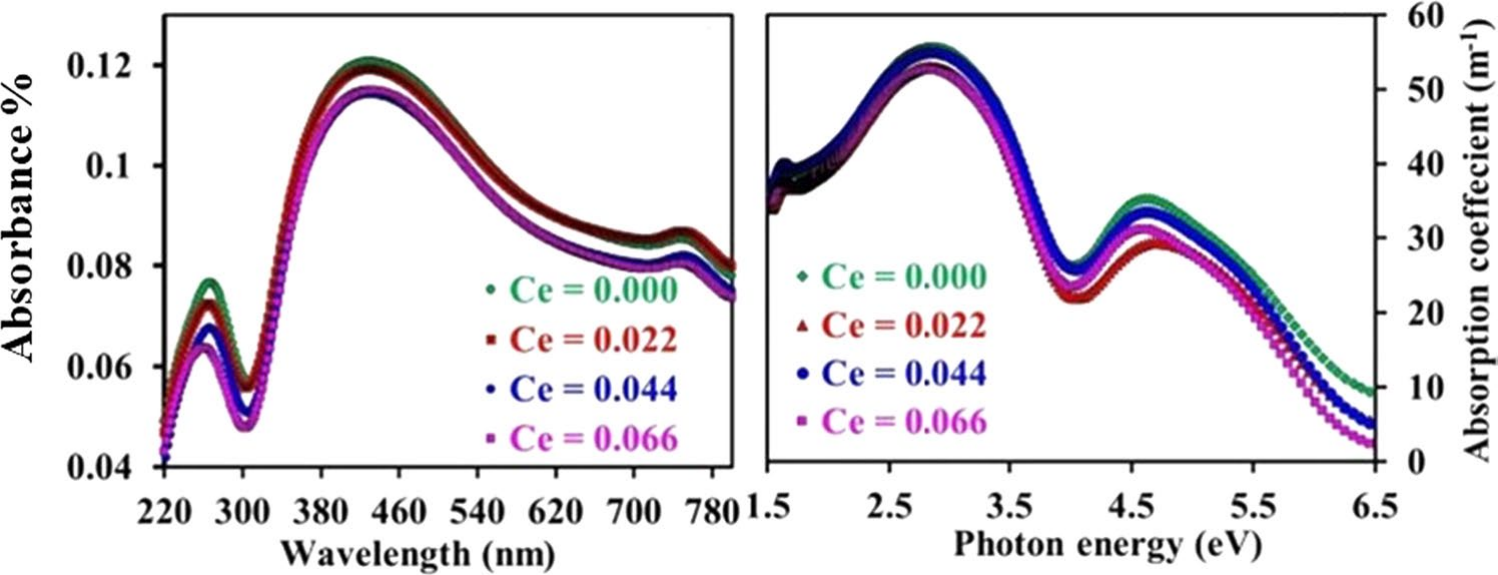
Absorption spectra and absorption coefficient of CNZ ferrite nanoparticles as a function of cerium ions content

The optical properties are directly related to their morphological, microstructural, and electronic characteristics, so the optical of property the material is important for different applications. Figure 7 shows the optical absorption spectra of CNZ spinel ferrite nanoparticles as a function of cerium rare earth ions content (*x*) measured by Uv–Vis spectroscopy at room temperature. It can be seen that the absorption spectra have the same trend for all the samples. From Fig. 7, it can be illustrated that no significant effect on the locations of the absorbance peaks by varied cerium ions content (*x*). As can be cleared from Fig. 7, the absorbance spectrum gives two strong absorption bands around 270 nm and 400 nm in the Uv range for all the investigated CNZ ferrite nanoparticles compounds due to the electronic transitions occurring from the O^2−^ valence band to Fe^3+^ conduction band [55, 56]. From Fig. 7, it can be also seen the relationship between the absorption coefficient (*α*) and photon energy (hν) of CNZ spinel ferrite nanoparticles as a function of cerium rare earth ions content. The two peak values of α are observed in UV and visible regions which indicate two values of the optical energy band gap. Moreover, it is apparent from Fig. 7 that there is a strong correlation between α and Ce ions in CNZ spinel ferrite compounds, where the value of absorption coefficient (*α*) decreases and as a result, the values of optical gaps increase with the increase of cerium ions in the fabricated samples.

**Fig. 8:**
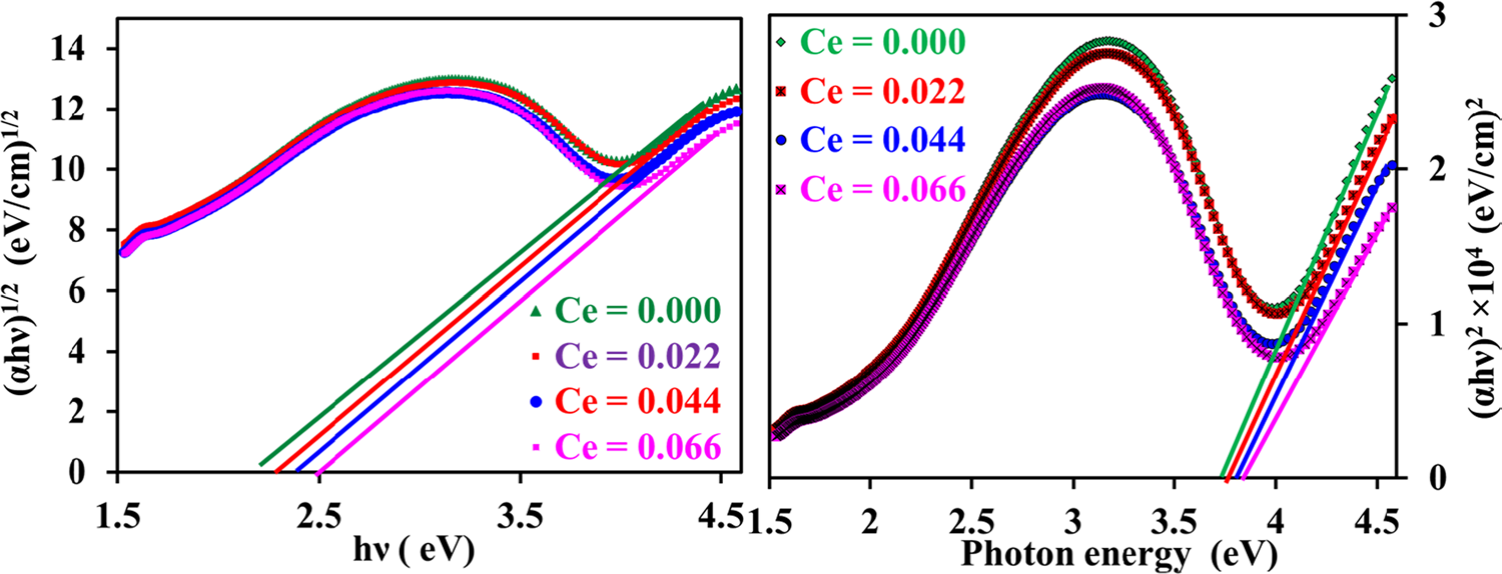
** a** (αhν)^1/2^ and **b** (αhν)^2^ versus the photon energy (hν) of CNZ ferrite nanoparticles a function of cerium ions content

To determine the direct (*E*_gdir._) and indirect (*E*_gind._) band gaps of the investigated samples, (*αhν*)^2^ and (*αhν*)^1/2^ were plotted against photon energy (*hν*), and its variation is shown in Fig. 8. Values of *E*_gdir._ and *E*_gind_. optical band gaps were estimated by intercepting the *x*-axis where the linear portion of the graphs was extrapolated and recorded in Table 3. From Table 3, it can be noticed that the band gap values are falling into the ranges (3.75–3.88 eV) and (2.1–2.5 eV) for direct and indirect bandgap, respectively. Further, it is observed that the values of both Eg dir. and Eg ind band gaps are increased with the increase in Cerium ions content (*x*). A similar result for direct optical band gap was observed in other spinel ferrite systems [57, 58]. The increase in *E*_gdir._ and* E*_gind._ may be attributed to the promotion of energy level and to the interface defects in CNZ spinel ferrite nanoparticles with the increase in the concentration of cerium rare earth ions (*x*). Moreover, this may have arisen due to inducing additional sub-band optical gap energy levels as a result of the introduction of the cerium rare earth ion in Ni–Zn spinel ferrite compounds [59]. Hence, by introducing cerium rare earth ions in Ni–Zn spinel ferrite the optical band gap can be tuned. The concentration of cerium ions (*x*) can be used to engineer the optical band gap for potential applications.

### Raman spectroscopic analysis

**Fig. 9:**
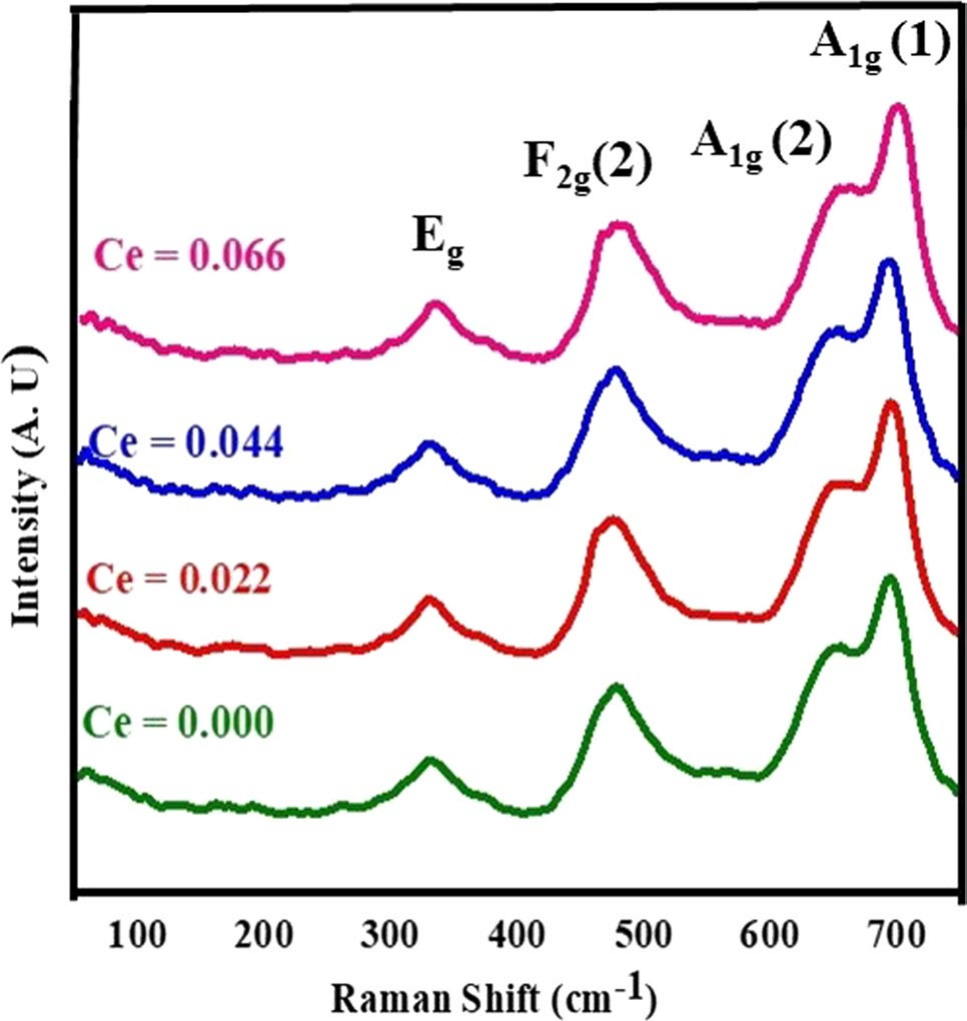
Raman spectra of CNZ ferrite nanoparticles as a function of cerium ions content

Raman Spectroscopy is a powerful examination instrument that provides excellent information via the structural properties of nano-spinel ferrite compounds. Figure 9 shows Raman spectra of CNZ spinel ferrite nanoparticles as a function of cerium rare earth ions content observed in the range of 0–800 cm^−1^ at room temperature and the vibrational modes data registered in Table 4. From Table 4, the Raman spectra illustrate four distinct-active modes (*E*g, F2g(2), A1g(2) and A1g(1)) for CNZ investigated samples. The symbols *A*, *E*, and *F* for the Raman modes denote one, two, and three-dimensional representations, respectively, and g stands for the symmetry around the reflection center [60]. Nickle zinc spinel nanoparticle ferrite compounds exhibit inversion of the cations between the tetrahedral and octahedral sites. The maximum intensities of the four mentioned first-order Raman spectroscopic peaks of CNZ ferrite nanoparticles were observed at about (330, 475, 650, and 695) cm^−1^, respectively, which means that the studied samples have a cubic spinel structure. The shifting in these four active modes peaks may depend on the redistribution of cations in the different sites of the ferrite compound structure with the cerium ions content. In addition, the substitution of cerium rare earth ions with big ionic radius in the investigated nickel–zinc ferrite nanoparticles modifies the Raman modes as a result of modifying the structural parameters such as crystallite size, lattice parameter, and bond lengths [61].

**Table 4:** The vibrational modes based on Raman spectra of CNZ spinel ferrite nanoparticles as a function of cerium ions content

*A*_g_ (1) (wm^−2^)	*A*_g_ (1) (cm^−1^)	*A*_g_ (2) (wm^−2^)	*A*_g_ (2) (cm^−1^)	*F*_2g_ (2) (wm^−2^)	*F*_2g_(2) (cm^−1^)	*E*_g_ (wm^−2^)	*E*_g_ (cm^−1^)	Ce (*x*)
220	695	174	647	154	481	95	330	0.000
231	697	177	650	162	480	93	331	0.022
242	695	194	648	175	481	115	330	0.044
251	697	195	650	179	480	117	331	0.066

**Fig. 10:**
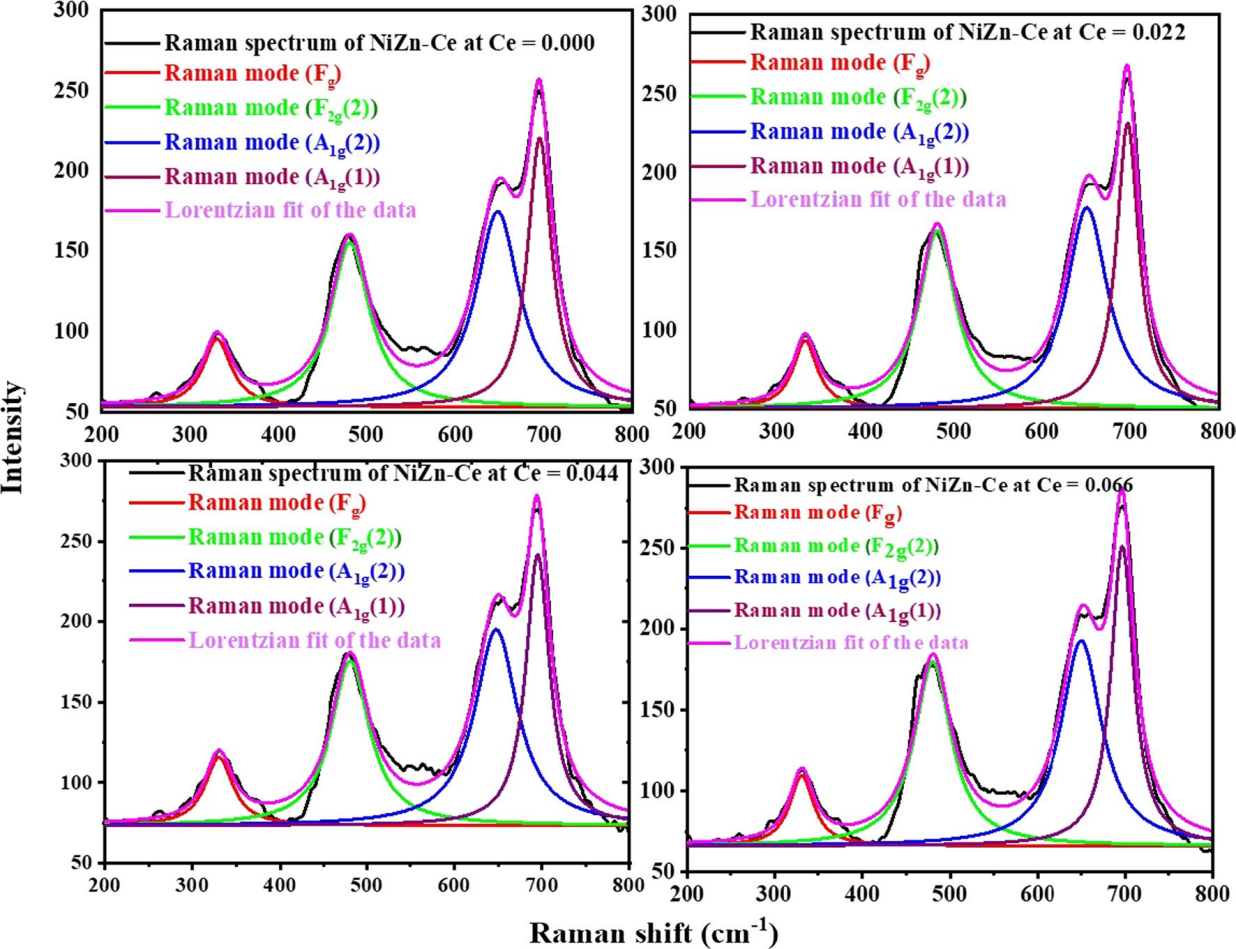
Lorentzian function fitting and Raman modes curves of Raman spectra of CNZ ferrite nanoparticles as a function of cerium ions content

We analyzed Raman spectra of CNZ spinel ferrite nanoparticles that fit a least square Lorentzian line shape. Therefore, Fig. 10 shows the best agreement of the experimental data curve with theoretical data via the Lorentz oscillation model. The strongest visual range for the samples examined at approximately 695 cm^−1^ is consistent with a similar study reported [62]. In the cubic structure of spinel ferrite, the active Raman modes above 600 cm^−1^ mostly preserved the symmetrical motion of oxygen ions at the tetrahedral sites within the spinel lattice [63–67], so the modes at around 650 and 695 cm^−1^ can be attributed to the splitting of Ag1 into A1g(2) and A1g(1) symmetries. This can be attributed to the random occupation of the tetrahedral sites by Zn^2+^, Ni^2+^ and Fe^3+^ cations and probably leads to slight differences in frequencies, which implies the creation of observed double vibrational peaks for A1g. A1g(2) Raman mode is associated with Zn–O and Fe–O bonds while A1g(1) mode represents the interaction of Ni–O and Fe–O bonds at tetrahedral sites [68, 69]. These two modes at about 650 and 695 cm^−1^ correspond to the A1g mode. The A1g band of normal spinel ferrite compounds ZnFe_2_O_4_, which occurs at t 664.97 cm^−1^ and at t 647 cm^−1^ and the A1g-band of the reverse NiFe_2_O_4_ spinel, which is found at position 698 cm^−1^ [70]. The oxygen atoms move near and far from the tetrahedral site (*A*) along the direction of the bonds, and at the same time, the tetrahedral cations do not move during this vibration. The Raman mode *E*g is due to the symmetric bending of oxygen ions concerning the cations of the (*B*) octahedral sites as it is closely related to the B–O bond distance [71]. When adding rare earth cerium ions to the Ni–Zn ferrite nanoparticle samples, they are preferable to enter the octahedral (*B*) sites at the expense of the ferric ions, which move to the tetrahedral (*A*) sites and turn into binary valence (ferrous ions), and this leads to a redistribution of nickel and zinc ions between *A* and *B* sites in order to maintain the electrical charge equilibrium of the formed ferrite compounds. The lower frequency modes (*E*g, and F2g(2)) than A1g signify the character of the octahedral (*B*) sites [63, 64]. Mode F2g(2) at about 450 cm^−1^ represents the movement of metal and oxygen ions bonds in opposite directions along one direction of the lattice or asymmetric motion of oxygen ions at octahedral sites. Table 1 indicates that the increase in the concentration of rare earth cerium ions in nickel–zinc ferrite nanoparticles and the redistribution of cations in the A and B sites according to that, does not affect the values of the frequencies of the Raman spectra bands, but does affect the relative intensity of those bands. The intensities of Raman modes at tetrahedral (*A*) sites higher than those of Raman modes at octahedral (*B*) sites may be a result of M–O bonds at *A* and *B* sites where every oxygen ion is bound with three cations at the B site and only with a single cation at the *A* site. All positions of Raman modes illustrate no dependence on the cerium ions content (*x*) but their intensities increase with increasing (*x*) which may be due to increasing the lattice parameter and the crystallite size with (*x*).

### Dielectric and electrical properties

**Fig. 11:**
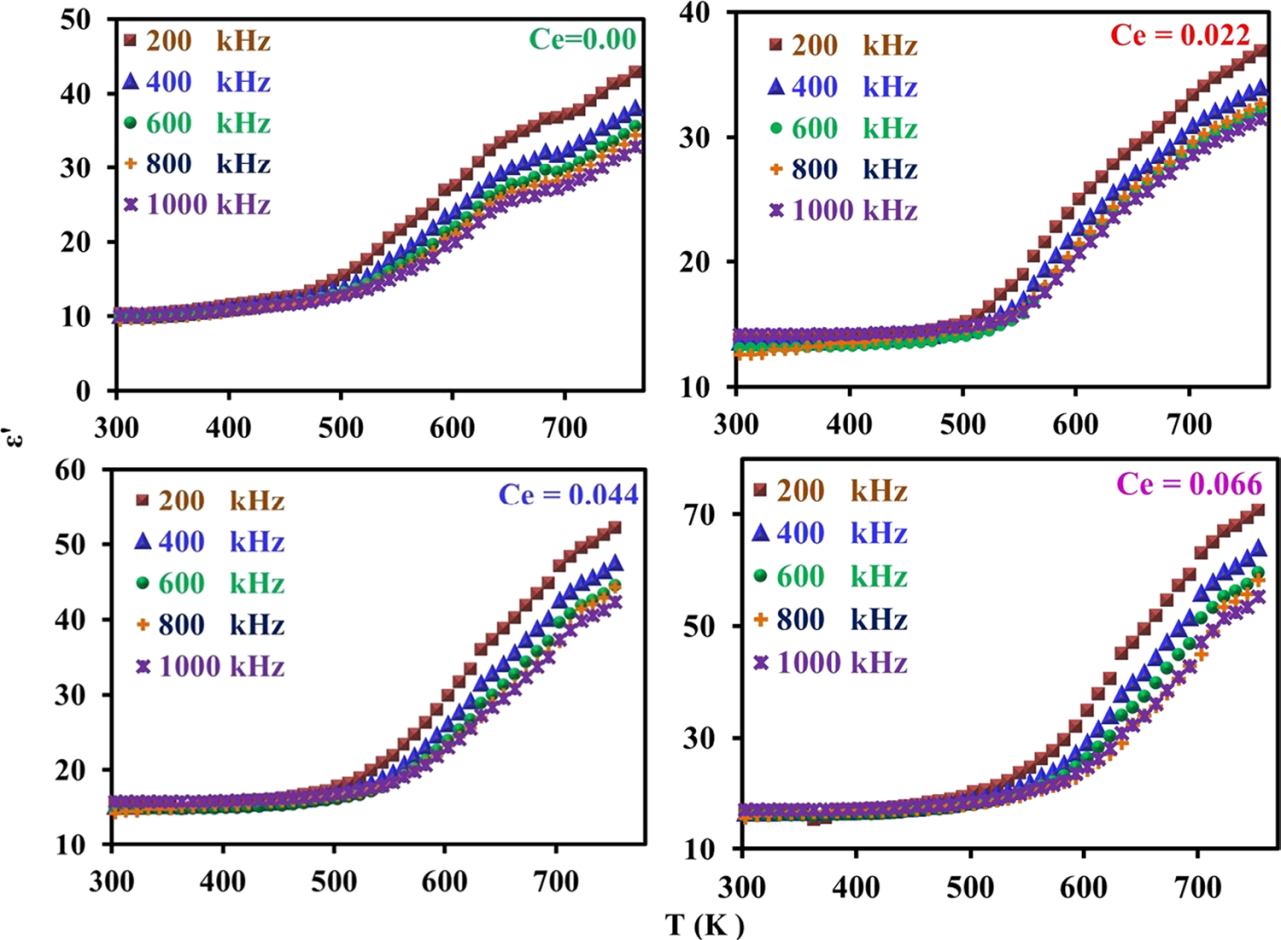
Dielectric constant (*ε*^/^) of CNZ ferrite nanoparticles samples versus absolute temperature at different frequency as a function for cerium ions content (*x*)

Figure 11 represents the typical curves for the relation between dielectric constant (*ε*/) and absolute temperature at different frequencies as a function of cerium ions content (*x*) in CNZ system samples. The curves in the figure show that all the investigated CNZ samples have the same character where *ε*/ generally increases with increasing both absolute temperature and cerium ions content (*x*). In addition, from these curves, it can be noticed that the relaxation peak at 660 K for the CNZ sample without cerium ions, and this peak disappears for the rest of CNZ ferrite samples doped with cerium ions in the studied range of temperature (300–770) K. It can be expected that the relaxation peak raising at temperatures higher than our study range may be due the heavy atomic mass of cerium ions. Moreover, the high thermal stability of CNZ ferrite compounds due to the high bond energy of Ce–O as compared to that of Fe–O may be lead to displacement in the dielectric constant relaxation peak toward higher temperatures.

The temperature assists in the liberation of localized dipoles, i.e., the number of free dipoles increases to reach the maximum with increasing temperature, whereas the ac field works to align them in its direction, which leads to an increase of *ε*^/^ up to the relaxation peak. With further increasing temperature additional localized charges are liberated from their ions and the aligned dipoles in the direction of the electrical field increase; hence the dielectric constant continues to increase. From the curves of *ε*^/^ vis *T* (K) it can be seen that έ become frequency dependent where in the high-temperature range, the orientational, interstitial, and hopping polarizations can participate in the relaxation process. In addition, all curves in the figures are divided into two regions, the first region begins from room temperature to ~ 530 K, in this region, the thermal energy given to the samples has no ability to free the localized dipoles, and the second region above ~ 530 K, in this region, the thermal energy is sufficient to liberate more localized dipoles, and at the same time, the applied alternating electric field pushes them to align it in its direction.

**Fig. 12:**
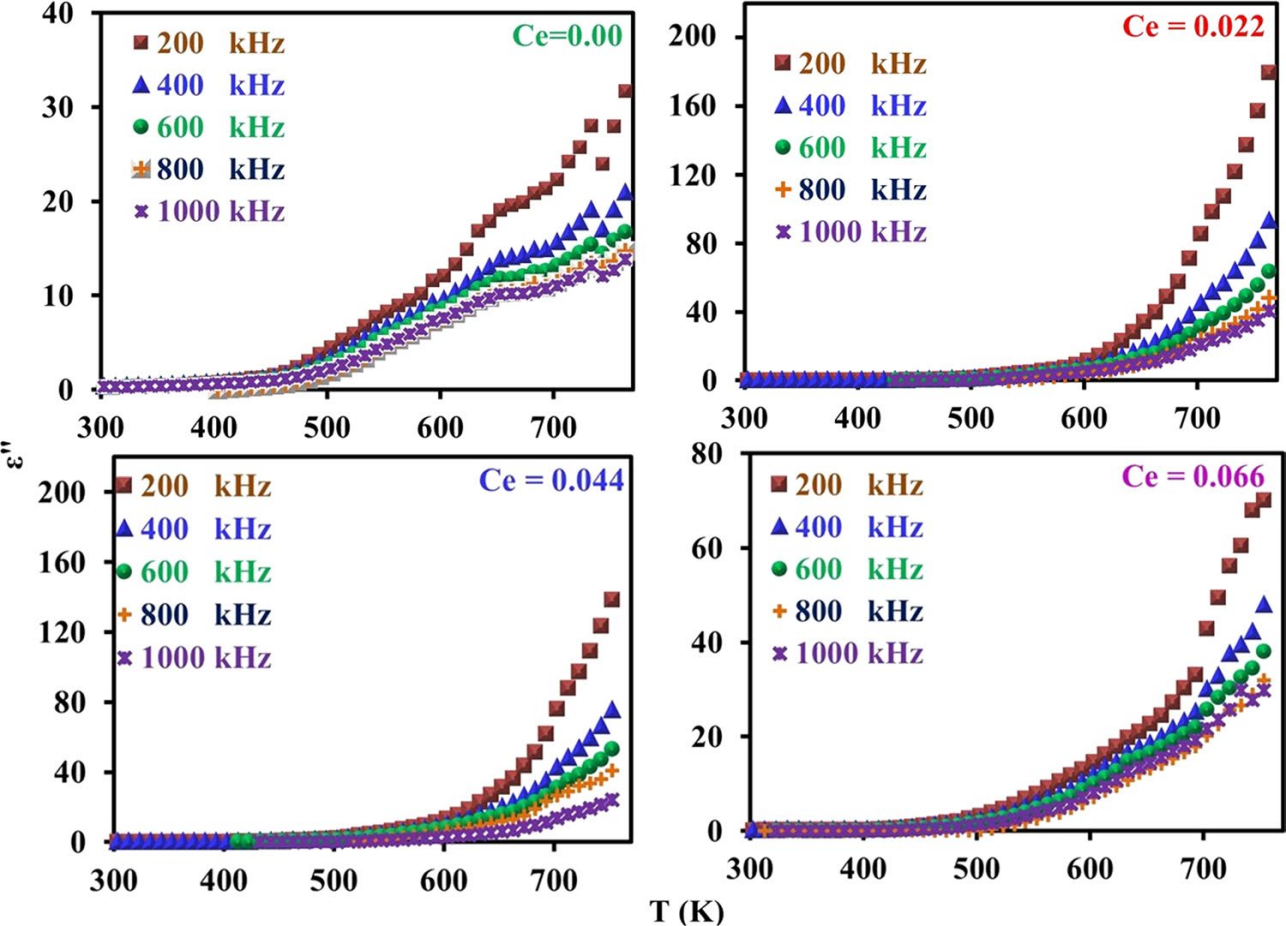
Dielectric loss factor (*ε*^//^) of CNZ ferrite nanoparticles samples versus absolute temperature at a different frequency as a function for cerium ions content (*x*)

**Fig. 13:**
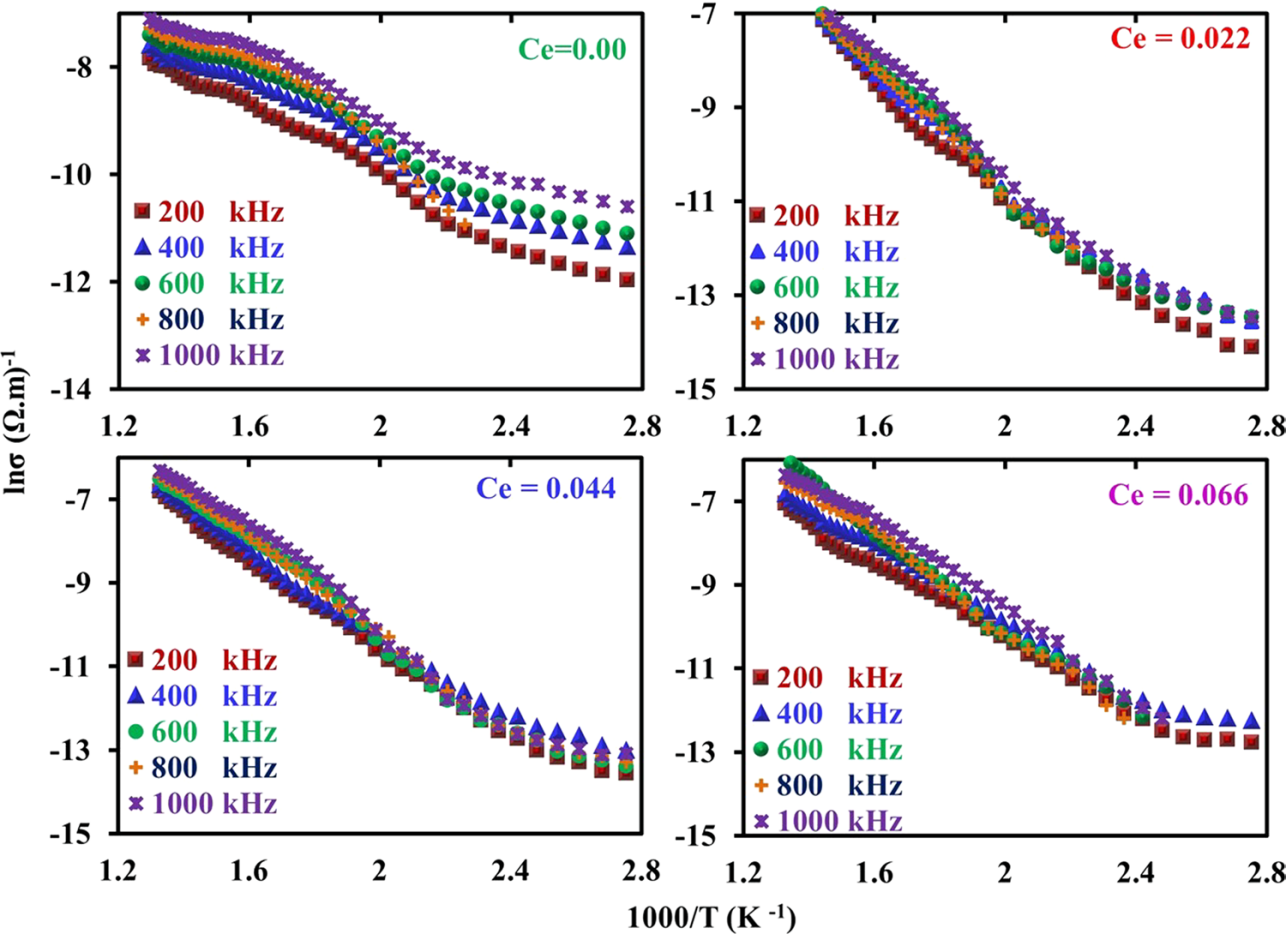
ln(*σ*) of CNZ ferrite nanoparticles samples versus inverses absolute temperature at a different frequency as a function for cerium ions content (*x*)

**Fig. 14:**
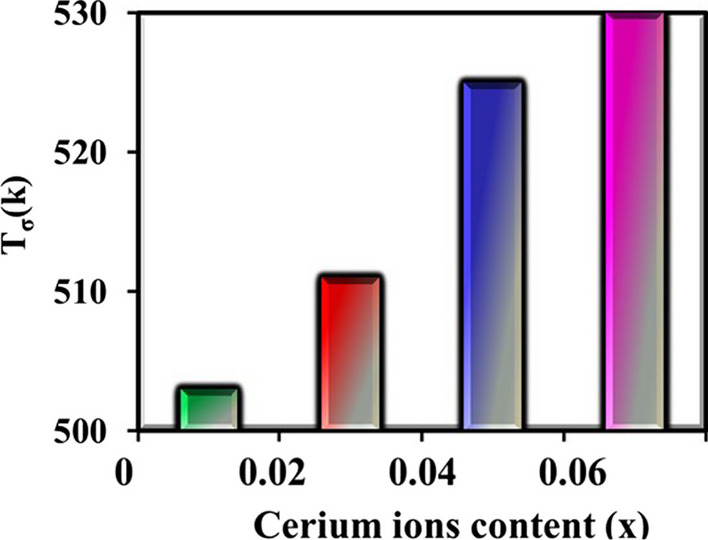
Transition conductivity temperature (*Tσ*) of the CNZ ferrites nanoparticles as a function of cerium ions content (*x*)

The curves of Fig. 12 show the dielectric loss factor (*ε*^//^) vis absolute temperature at different frequencies as a function of cerium ions content (*x*). From this figure, it can be noticed that all the CNZ samples have the same mode where *ε*^//^ generally increases with increasing absolute temperature and decreases with frequency. With a decrease in the frequency of the applied electric field, the response of the electric dipoles increases and moves in parallel with the electric field, and at the same time, with an increase in temperature, the dispersion of the dipoles increases, which increases the energy loss and therefore increases the dielectric loss. Figure 13 illustrates the relation between ln(*σ*) and reciprocal absolute temperature at different frequencies as a function of cerium ions content (*x*). It can be seen from the curves of the figure that the electrical conductivity has a semiconductor nature where it increases with temperature increases. The conductivity of ferrites is primarily studied by the role of grain boundaries (GB) since ferrites are considered to be composed of conductive grains separated by resistive grain boundaries (GB) based on Koops's theory [72–74]. The conduction in spinel ferrites occurs based on charge carriers hopping between the same element ions in different valence states [75, 76]. Hence, with the increase in temperature, thermal activation is awarded for charge carriers of the sample so the hopping process between the same ions with different valence in the CNZ sample increases. The weak regularity of the curves, which can be observed in the higher temperature is attributed to the higher thermal energy given to the charges of the sample and leads to the disruption of alignment for each other. The conduction mechanism in the two regions is different where the hopping electrons mechanism is the controller in the first region at low temperature whereas the hopping polaron mechanism at high temperature is dominant. The kink in all the curves in these figures indicates the transition temperature of CNZ ferrite samples where the sample transforms from a ferrimagnetic nature at the low-temperature region to a paramagnetic nature at the high-temperature region. The transition temperatures for the CNZ samples with different cerium ions content (*x*) are plotted in Fig. 14. The increase in transition temperature with the increase of cerium ions concentration may be due to the increase in the jumping distance between the same type of metal ions with different valences (Fe^3+^ and Fe^2+^ ions). The transition temperature value in this study for nickel–zinc ferrite samples doped with Ce is about 520 K, greater than 383 K for nickel–zinc ferrite samples doped with Yb and Gd, [26] and less than 600 K for nickel–zinc ferrite samples doped with La [27]. This means that the type of ground element added to the structure of nickel–zinc ferrite plays a significant role in changing its electrical and dielectric properties.

**Fig. 15:**
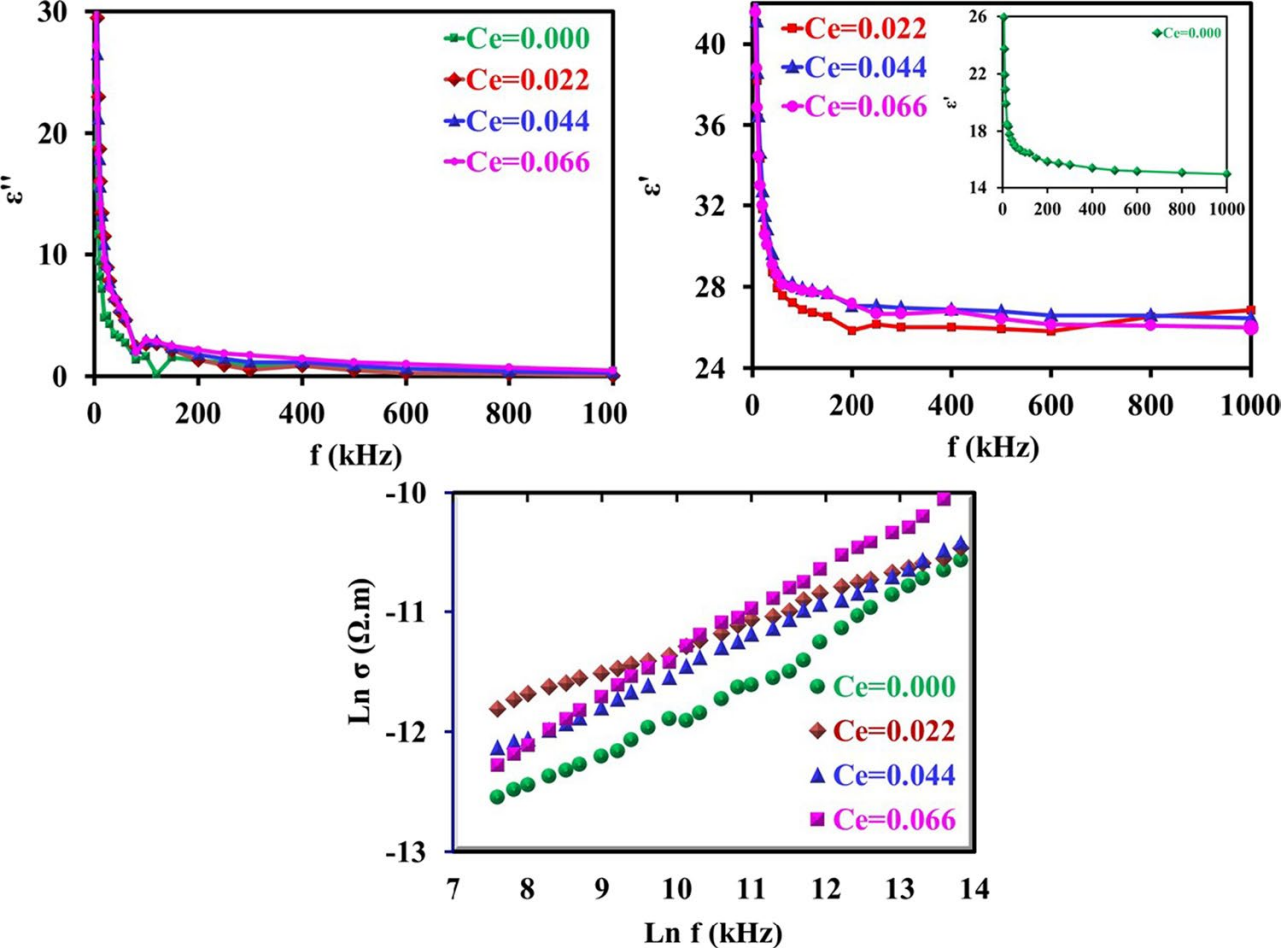
Dielectric constant (*ε*^/^), dielectric loss factor (*ε*^//^) and AC conductivity ln(*σ*) of CNZ ferrite nanoparticles samples versus frequency as a function for cerium ions content (*x*)

Figure 15 represents the dielectric constant (*ε*^/^), dielectric loss factor (*ε*^//^) and AC conductivity ln(*σ*) of CNZ ferrite nanoparticles samples versus frequency as a function for cerium ions content (*x*) the range from 1 to 1000 kHz at room temperature. The curves in the figure show that the dielectric constant (*ε*^/^) decreases with increasing frequency for all samples. This is suitable for high-frequency device applications [77–79]. It is observed that the conductivity increases with increasing concentration of cerium ions in the prepared CNZ samples and with the frequency. This is probably due to the affinity of iron ions as a result of the decrease in the length of the bonds in *A* and *B* sites, also a unit cell reduces and jump length for hopping of electrons decreases, with the increasing cerium ions content in the CNZ samples, as shown in Table 2. Moreover, the presence of cerium ions inside the lattice leads to an increase in the interfacial charge and the space charge polarizations as a result of the increase in the dislocation density as can be seen in Table 2, which enhanced the dielectric constant. This makes the CNZ compounds eligible for energy storage applications. It can be noticed that all the CNZ investigated samples exhibit a rapid decrease in the values of both *ε*′ and *ε*′′ with increasing frequency in lower frequencies. At higher frequencies, *ε*′ and *ε*′′ decrease slowly without any anomalous behavior, and then the decrease continues to reach constant values. This character of dielectric parameters can be explained on the basis of the hopping charge model and space charge polarization. The larger value of *ε*′ in the low frequencies zone is mainly due to the contributions of all types of polarizations including polarizations of interfacial and space charge which are due to the inhomogeneous structure as indicated by Maxwell–Wagner [80, 81]. The contribution of the charge carriers to *ε*′ decreases with the frequency increase. *ε*′ reaches a constant value in the high-frequency region attributed to the fact that above a specific frequency of the applied electric field, the electron hopping between iron ions cannot follow the fast oscillations in the applied field [54]. AC conductivity (*σ*_AC_) patterns of CNZ samples with the frequency are linear with respect to almost the entire range of frequencies as can be seen in Fig. 15. The behavior of the σ_AC_ can be assigned to the hopping of charge carriers between local states at octahedral sites (*B*), which confirm a small polaron type of conduction mechanism [82, 83]. An increase in the frequency of the applied field accelerates the hopping of charge carriers, and the hopping rate of charge carriers increases between Fe^2+^ and Fe^3+^ ions [84, 85] so the *σ*_AC_ of CNZ samples increases with increasing frequency. Moreover, we can realize from the figure that the *σ*_AC_ of CNZ samples increases with increasing concentration of cerium ions at the octahedral sites. The entry of cerium ions into the unit cell of the sample reduces the distance between the Fe^2+^ and Fe^3+^ ions that due to the reduction of the cation bonds (*L*_B_). Thus, it caused an increase in the jump rate of iron ions which play an essential role in increasing *σ*_AC_.

## Conclusion

CNZ ferrite system prepared by coprecipitation method. XRD analysis showed that CNZ samples with chemical formula Ni_0.55_Zn_0.45_Fe_2_Ce_*x*_O_4_ have monophase with cubic spinel structure for cerium ion content (*x*) from 0 to 0.066 and the solubility limit of cerium in the stoichiometric ratio of nickel and zinc ferrite is approximately 0.066 when prepared at preparation conditions of pH = 11 and sintering temperature = 1050°. SEM images exhibited that grains of the prepared CNZ ferrite compounds are fine, smooth, and semi-homogeneous spherical crystals. In addition, the particles become smaller, more perfect, and more homogeneous than that of the undoped Ni–Zn ferrite. FTIR spectra of CNZ ferrite nanoparticles were observed in the ranges 601.68 –593.97 cm^−1^ and 428.11–418.28 cm^−1^. FTIR peaks shift to lower values of the vibrational bands *V*_A_ and *V*_B_ with Ce^3+^ ions content (*x*) in Ni–Zn spinel lattice. The concentration of cerium ions (*x*) can be used to engineer the optical band gap for specific potential applications, where the values of direct and indirect optical band gaps are shown in a wide range from insulators to semiconductor materials (3.75–3.88 eV and 2.2–2.5 eV), respectively. The samples are semiconductor-like materials, where the AC conductivity increases with increasing temperature. The results show that the conduction mechanism depends on the cerium ions content (*x*). The conductivity transition temperature (*T*_σ_) increases with increasing cerium ions content (*x*). Ni_0.55_Zn_0.45_Fe_1.944_ Ce_0.066_O_4_ sample gave the best results, as the best homogeneity and least particle aggregation were obtained with the lowest value for the lattice constant, crystalline size, porosity, and the hopping lengths (*L*_A_) and [*L*_B_]. At the same time, this sample obtained the largest value of both specific surface area, optical energy gap, dielectric constant, and conductivity transition temperature (*T*_σ_). These results indicate that Ni_0.55_Zn_0.45_Fe_1.944_ Ce_0.066_O_4_ ferrite nanoparticles may be selected for optoelectronic, high-frequency and energy storage applications.

## Data Availability

All data generated or analyzed during this study were included in this published article, and if any data related to the current study is required, the corresponding author can be contacted.
